# Use of Cryopreserved Sperm of Grass Carp (*Ctenopharyngodon idella*) for Seed Production at the Hatchery Level of Bangladesh—A Need for Development of Germplasm Repositories

**DOI:** 10.1155/2024/9655558

**Published:** 2024-10-01

**Authors:** Shajjad Hossian, Md. Faijan Reza, Mohammad Matiur Rahman, Mariom, Mohammed Jahangir Alam, Md. Abdur Razzak, Md. Fazlul Awal Mollah, Terrence R. Tiersch, Md. Rafiqul Islam Sarder

**Affiliations:** 1 Department of Fisheries Biology and Genetics, Bangladesh Agricultural University, Mymensingh, Bangladesh; 2 Aquatic Germplasm and Genetic Resources Center, Louisiana State University Agricultural Center, Baton Rouge, Louisiana, USA

## Abstract

Grass carp (*Ctenopharyngodon idella*), a freshwater nonnative fish species, is a potential aquaculture candidate in Bangladesh. The seed of the species is produced in the hatcheries by hypophysation, but the quality of seedstock of grass carp is deteriorated due to inbreeding, negative selection, and interspecific introgression among fishes. To increase the availability of quality seed and best genetic traits of grass carp, this study dealt with finding suitable conditions of sperm cryopreservation protocols and evaluated the effectiveness of cryopreserved sperm through breeding trials of *C. idella*. A broodstock population was developed from fingerlings imported from China by the Bangladesh Department of Fisheries. Sperm was collected from hormone-induced mature males, with an estimated concentration of 2.4 ± 0.3 × 10^10^ cells/mL and a pH of 8.3 ± 0.2. Sperm motility was evaluated in different concentrations of NaCl solution. The highest motility (96% ± 1%) and longest motility duration (22 ± 1 min) were achieved at 0.4% of NaCl (128 mOsmol/kg). The toxicity of DMSO and methanol at concentrations of 5%, 10%, and 15% was evaluated for 5–40 min using Alsever’s solution and egg yolk citrate. The highest motility was achieved during 5 and 10 min of incubation with cryoprotectants at 5% and 10%. Alsever’s solution with 10% DMSO at 1:9 dilution with sperm produced the highest equilibration motility (93% ± 2%) and when cooled at 10°C/min yielded the highest postthaw motility (85% ± 3%). Fertilization of 24% ± 3% to 51% ± 2% and hatching of 18% ± 2% to 41% ± 2% were achieved by using cryopreserved sperm in six selected private hatcheries. The fertilization rate for fresh sperm sourced from hatchery-reared males was 64% ± 5% to 85% ± 3%, and the hatching rate ranged from 53% ± 6% to 74% ± 5%. Thus, the cryopreservation protocol of *C. idella* sperm was found to be feasible for fry production at commercial hatcheries, but further research is needed to improve the fertilization and hatching rates.

## 1. Introduction

Grass carp (*Ctenopharyngodon idella*) is a freshwater fish species indigenous to eastern Asia, mainly China and Eastern Siberia, particularly in the Amur River system. This species has been introduced worldwide as a biological method for controlling aquatic vegetation. Their primary food source is aquatic plants, making them essential for open water management in freshwater ecosystems as they are herbivorous. Also, among other Chinese carps, grass carp have been receiving worldwide attention as a source of food and are well-known for their delicious meat and substantial nutritional content [1]. Grass carp was introduced to Bangladesh from Hong Kong in 1966 and from Japan in 1979 for the purpose of controlling aquatic weeds and aquaculture [2].

The production and consumption of fish have significant effects on the economy and food security of Bangladesh. Fishes are the second-most valued agricultural crop in Bangladesh and provide millions of people with a vital source of livelihood and employment. Aquaculture production contributed 57.39% of the total production of the country, and it has continued to expand over the last three decades [3]. Exotic carps contributed 17.83% to aquaculture and 10.23% of total production [3]. Among the exotic carps cultured in Bangladesh, grass carp contributes 2.61% (0.071 million MT) of the total aquaculture production [3]. Aquaculture production has been substantially increased over the years due to production of seed in hatcheries. However, sustainable production has not been ensured, because environmental and anthropogenic threats are continuing to reduce production. Limited availability of quality seed of grass carp and inadequate supply to farmers are major problems leading to decreased production. In addition, genetic management is compromised by inbreeding, interspecific hybridization, genetic drift, and negative selection during seed production in hatcheries. Consequently, stocked fry often display slow growth, high mortality, and disease susceptibility. These existing genetic problems can be effectively mitigated through the cryopreservation of sperm and subsequently establishment of male germplasm repositories.

Fish germplasm repositories are specialized facilities or institutions that store and preserve genetic material (sperm, eggs, embryos, and tissue samples) from various fish species. These repositories allow storage of genetic material for long periods and provide a readily accessible source of gametes that can be transported easily [4]. The primary purpose of fish germplasm repositories is to conserve the genetic diversity of wild fish populations and improve the genetic constituents of domesticated fish for the long-term benefit of breeding programs at the hatchery level, brood quality improvement, and ultimately aquaculture production enhancement. A cryopreserved male gamete repository is an effective tool for preserving and refining the genetic resources of commercial aquaculture species [5, 6], and their use in commercial could resolve the existing problems at the hatchery level. Cryopreservation is an important ex situ method of germplasm conservation, and the Food and Agriculture Organization of the United Nations has endorsed it as a major strategy for conservation of fish resources [7]. It has been studied around the world, and sperm cryopreservation protocols have been developed for more than 200 species of finfish and shellfish [8] including endangered fish species [9]. Numerous advantages of cryopreservation in fisheries include conserving genetic diversity, selective breeding, hybridization, and a reliable supply of gametes for hatchery seed production or laboratory investigation [10, 11].

In Bangladesh, research on fish sperm cryopreservation was started in the Department of Fisheries Biology and Genetics, Bangladesh Agricultural University (BAU), in 2004, and preliminary protocols for cryopreservation of sperm have been developed for Indian major carps (IMCs), exotic carps, cat-fishes, and endangered fish species [12–14]. The first sperm cryopreservation work on grass carp in Bangladesh focused on protocol development in laboratory settings [15]. But no significant study has been conducted on the activation solution, toxicity of cryoprotectant, equilibration time, and cooling rate of *C. idella* sperm. Besides, no breeding was conducted using cryopreserved sperm at hatchery level of this species. Therefore, a study was needed to see whether it is feasible to use cryopreserved sperm in stakeholder installations (hatcheries) to produce quality seed and future broodstock of *C. idella*. A successful completion of this endeavor will ultimately call for the establishment of cryogenic germplasm repositories.

## 2. Materials and Methods

### 2.1. Rearing of Broodstock.

A broodstock population of grass carp was developed by collecting and rearing imported fingerlings from China by the Department of Fisheries (Brood Bank Establishment Poject 3^rd^ Phase in 2019) with a view to replenish the stock as the existing population has genetically deteriorated by closed mating, interspecific hybridization, and genetic drift. Fingerlings were reared in ponds while maintaining a stocking density of 1000–1200 kg/ha in the vicinity of Fisheries Faculty building, BAU. The fish were provided with supplementary feed (Pushtiraj Feed, ARMAN Feeds and Fisheries Limited, Bangladesh) containing a minimum 25% protein twice a day at 4%–5% of their body weight. In addition, green grass and banana tree leaves were provided to the fish. Moreover, addition of underground water, liming (250 g/decimal/month), monitoring of fish health, and water quality on a regular basis were also performed during the experimental period.

### 2.2. Collection of Sperm.

Mature males were collected from the broodstock ponds and selected on the basis of phenotypic characteristics such as rough pectoral fin (the presence of tubercles is a visual indicator of readiness) and flat abdomen. For conditioning, selected brood fish were held in a circular tank for about 6 h prior to hormone injection and a constant water flow was provided to ensure proper aeration. A single dose of carp pituitary gland (PG) extract (imported from India through a local supplier) at 2 mg/kg body weight was injected intramuscularly on the dorsal side below the lateral line with a 1 mL syringe, and after 6 h, sperm samples were collected by abdominal stripping. Before collection of sperm, excess moisture, urine, gut exudate, and mucus were wiped from the genital region with absorbent paper. To avoid contamination by urine or water, a gentle pressure was applied to the abdomen to release a small amount of sperm before final collection. Concentrated whitish milt was collected in autoclaved 1.5 mL tubes (Eppendorf, Biologix Group Limited). Sperm samples were labeled and placed immediately on ice for storage.

### 2.3. Estimating Sperm Quality and Motility.

The quality of fresh sperm was examined (within 5 min of collection) by use of a light microscope (Olympus, Model CX41 and DP22, Japan). A 2 μL sperm sample was diluted with 18 μL of distilled water and placed on a glass slide. The video screen display of the microscope at 10x and 20x magnification was used to estimate sperm motility immediately after being mixed. Five fields from the video screen display were selected randomly (both for fresh and cryopreserved sperm), and the motile sperm were counted directly by observing the video screen based on their active movement. Sperm were considered motile if they presented any type of movement (progressive or nonprogressive). Each motility assessment was performed in triplicate. Motility was estimated by the same evaluator in all cases. The entire procedure was executed at room temperature (±25°C). The motility value was expressed as the percentage, and the mean value was calculated. In addition, sperm concentration, pH, and motility duration were determined. The pH of sperm was measured using litmus paper (Rankem Indicator Paper, Ranbaxy Fine Chemicals Limited, New Delhi, India). Sperm concentration was assessed by use of a hemacytometer (Precicolor, HBG, Germany) counting and expressed as the number of cells × 10^10^/mL. This value was used to identify the sperm–egg ratio during fertilization trials.

### 2.4. Selection of Suitable Activation Solution.

To select an activation solution, motility and motility duration of sperm were tested at various concentrations of NaCl [16, 17]. Twelve graded dilutions of NaCl (0.1% to 1.2%) corresponding to osmotic pressures ranging from 48 to 383 mOsmol/kg (measured using a Vapor Pressure Osmometer (VAPRO 5600), Wescor, ELITechGroup Inc., USA) were prepared by dissolving NaCl (Merck Life Science Private Limited, Mumbai, India) in distilled water for each dilution. As described above, 2 μL of sperm was placed on a glass slide, and 18 μL of each NaCl dilution was added to activate the cells. The percentage of motility and motility duration of activated sperm was determined.

### 2.5. Determination of Suitable Cryoprotectant Solutions.

Multiple cryoprotectant treatments were evaluated to select candidates prior to freezing studies [16, 18]. In the current study, two extenders, Alsever’s solution and egg yolk citrate, and two cryoprotectants, dimethyl sulfoxide (DMSO) and methanol, were tested. Each cryoprotectant was mixed with extenders to prepare final concentrations of 5%, 10%, and 15% in the diluents. Milt was diluted with diluent at a ratio of 1:9 for Alsever’s solution and 1:4 for egg yolk citrate solution. For each concentration, 1–2 μL of the milt–diluent solution was placed on a glass slide, and sperm movement was tracked with a photography system (Olympus, Model CX41 and DP22, Japan) by observing video screen display at 10x and 20x magnification for 5–40 min at 5-min intervals.

### 2.6. Determination of Suitable Diluent and Sperm–Diluent Ratio.

Two extenders, Alsever’s solution (pH, 7.9 and osmolality, 240 mOsmol/kg) and egg yolk citrate (pH, 6.7 and osmolality-222 mOsmol/kg), and two cryoprotectants, DMSO and methanol, were used to determine the suitable dilution ratio. Alsever’s solution was formulated by dissolving 0.4 g of sodium chloride and 0.8 g of sodium citrate in 100 mL of distilled water. For the preparation of egg yolk citrate, initially, 0.4 g of sodium chloride and 0.3 g of sodium citrate were dissolved in 100 mL of distilled water to prepare a buffer solution. Subsequently, in a volumetric ratio of 1:4 (egg yolk to buffer), 20 mL of egg yolk were combined with 80 mL of the prepared buffer solution to yield the final solution. Equilibration and postthaw motility of sperm were evaluated to select the diluent and sperm–diluent ratio for cryopreservation. The diluents were prepared by mixing 10% of cryoprotectants with 90% of extenders (%v/v). Sperm samples were diluted with three different ratios, 1:9, 1:12, and 1:15 (sperm:diluent), for Alsever’s solution, and 1:4, 1:6, and 1:9, for egg yolk citrate. The sperm: diluent (activation solution) ratios were chosen in accordance with the previous study on grass carp [15], silver carp [13], and rohu [12] sperm cryopreservation. Diluted sperm samples were equilibrated on ice for 15 min after mixing. Sperm motility was evaluated at the end of equilibration as described above.

Two hundred thirty microliter (230 μL) of diluted sperm was loaded into 250-μL French plastic straws with a micro-pipette. The straws were manually sealed using heated crucible tongs. The filling and sealing straws were completed during the equilibration period (15 min). The sealed straws were loaded into a cryochamber which was immersed in a cryobath prefilled with liquid nitrogen. A programmable computerized freezer (CL-3300, which consists of a temperature controller, cryochamber, and cryobath. The chamber stands directly in liquid nitrogen in the cryobath and is connected to the controller which regulates the temperature of the specimens), operated by Cryogenesis software version 4 (Cryologic Pty. Ltd., Australia, 1998 and 1999), and was used to lower the temperature of the samples from 4°C to −80°C employing a single-step freezing procedure with a gradual decline at a rate of 10°C/min [19]. For storage, the frozen straws were moved to a liquid nitrogen-filled surgical tray where they were placed in aluminum cryocanes (Thermo Fisher Scientific), and they were transferred to a liquid nitrogen dewar (−196°C). The whole process was accomplished within 1 min. For observing postthaw motility of cryopreserved sperm, frozen straws were retrieved from the dewar flask containing liquid nitrogen by dipping the whole canister at a minimally visible level and pulling it out of liquid nitrogen with forceps and thawed at room temperature (±25°C) for 1 min. The straws were cut at both ends, and 2 μL of thawed sperm was placed on a glass slide for motility estimation under a microscope.

### 2.7. Determination of Suitable Equilibration Time.

The sperm samples were equilibrated at four time periods (5, 10, 15, and 20 min) at 4°C, and motility was evaluated at each time. The diluents were prepared by mixing 10% cryoprotectants (DMSO and methanol) with 90% extenders (Alsever’s solution and egg yolk citrate). Sperm samples were diluted with the diluents at the ratio of 1:9 (sperm:diluent) for Alsever’s solution and 1:4 for egg yolk citrate. The equilibration motility of the diluted sperm was evaluated at the end of each equilibration period with three replicates.

### 2.8. Determination of Suitable Cooling Rate.

To determine the effect of cooling rate on postthaw motility of sperm, samples were frozen with three different rates (5, 10, and 15°C/min). Samples were processed with two extenders (Alsever’s solution and egg yolk citrate) and two cryoprotectants (DMSO and methanol) and equilibrated for 15 min. The diluents were prepared by mixing 10% cryoprotectants with 90% extenders. Sperm dilution was maintained at 1:9 (sperm:diluent) for Alsever’s solution and 1:4 for egg yolk citrate and cooled from 4 to −80°C using the one-step freezing protocol described above. The postthaw motility of cryopreserved sperm was evaluated with three replicates.

### 2.9. Effects of Cryopreserved Spermatozoa on Fertilization and Hatching Rates.

To determine the effects of cryopreserved spermatozoa on fertilization and hatching rates, breeding trials were performed in six private hatcheries in four regions of Bangladesh: Sotota Matshyo Prozonon Kendra and Fishery in Mymensingh; Matri Fish Hatchery and Integrated Farm, Mukteshwari Fish Hatchery, Sonali Fish Hatchery, and Matsho Kanon Hatchery and Nursery in Jashore; and Motsha Bangla Fish Hatchery in Jhalokathi. To induce ovulation, female fish were administered two doses of PG hormone injection according to their body weight (the first dose was 2 mg/kg body weight, and the second dose was 4 mg/kg body weight) at a 6-h interval. Ovulated eggs were collected from hatchery-origin females by stripping after 6 h of second hormone injection. Collected eggs were divided into plastic bowls using a teaspoon and were fertilized with cryopreserved and fresh sperm. The cryopreserved sperm of China origin stock were carried to the selected hatcheries in 10-L liquid nitrogen dewars. Extra liquid nitrogen was carried to the hatcheries in another 10-L dewars for supporting the cryopreserved straws if needed. Before fertilization, the motility of frozen sperm will be checked under a microscope.

For the treatment group, frozen sperm was thawed for 1 min at room temperature and gently mixed with eggs with a hen feather. Pooled sperm from 30 straws was mixed with three teaspoons of eggs (three teaspoon ≈ 15.000 eggs), and 1–2 mL of Alsever’s solution was added during mixing. A similar number of eggs from the same donor female were also fertilized with fresh sperm from hatchery-originated males to estimate egg quality. No attempt was made to standardize the sperm numbers, but each treatment received an excess of sperm. After fertilization, eggs of both groups were incubated separately in two incubators maintaining a constant water flow. At 5–7 h of fertilization, about 100 eggs were collected at least three times from each incubator, and fertilized and unfertilized eggs were counted separately to estimate the fertilization rate. The fertilized eggs appeared clear and transparent, while unfertilized eggs appeared opaque with a white chorion. The fertilization rates were calculated by using the following formula:

$$\text{Fertilization}\;\text{rate}\;(\%)=\frac{\text{No.}\;\text{of}\;\text{fertilized}\;\text{eggs}}{\text{Total}\;\text{no.}\;\text{of}\;\text{eggs}}\times 100.$$

Within 18–24 h of fertilization, eggs began to hatch at a temperature of 26 to 28°C. The hatchlings of the control and treatment incubators were sampled and counted. The hatching rates were calculated by using the following formula:

$$\text{Hatching}\;\text{rate}\;(\%)=\frac{\text{Total}\;\text{no.}\;\text{of}\;\text{hatchlings}}{\text{Total}\;\text{no.}\;\text{of}\;\text{eggs}}\times 100.$$

The hatchlings were counted and moved to nursery ponds for rearing.

### 2.10. Statistical Analysis.

Data for activation, cryoprotectant toxicity, sperm–diluent ratio, equilibration time, and cooling rate were presented as the percentage of motile cells and were converted by arcsine transformation for statistical analysis. One-factor analysis of variance of the Statistical Package for the Social Science (SPSS version 23) was used for analyzing the data of activation, toxicity, sperm–diluent ratio, equilibration time, and cooling rate, and the means were separated by Duncan’s multiple range test at 5% level of significance.

## 3. Results

### 3.1. Initial Evaluation of Sperm Quality.

Sperm was collected from the males by stripping. The concentration of fresh sperm was 2.4 ± 0.3 × 10^10^ cells/mL, and the pH was 8.3 ± 0.2. The motility of fresh sperm was estimated as 90% ± 1%. During motility observation, sperm displayed forward (i.e., motility) and Brownian movement.

### 3.2. Effect of Osmotic Pressure on Sperm Motility Activation.

**Figure 1:**
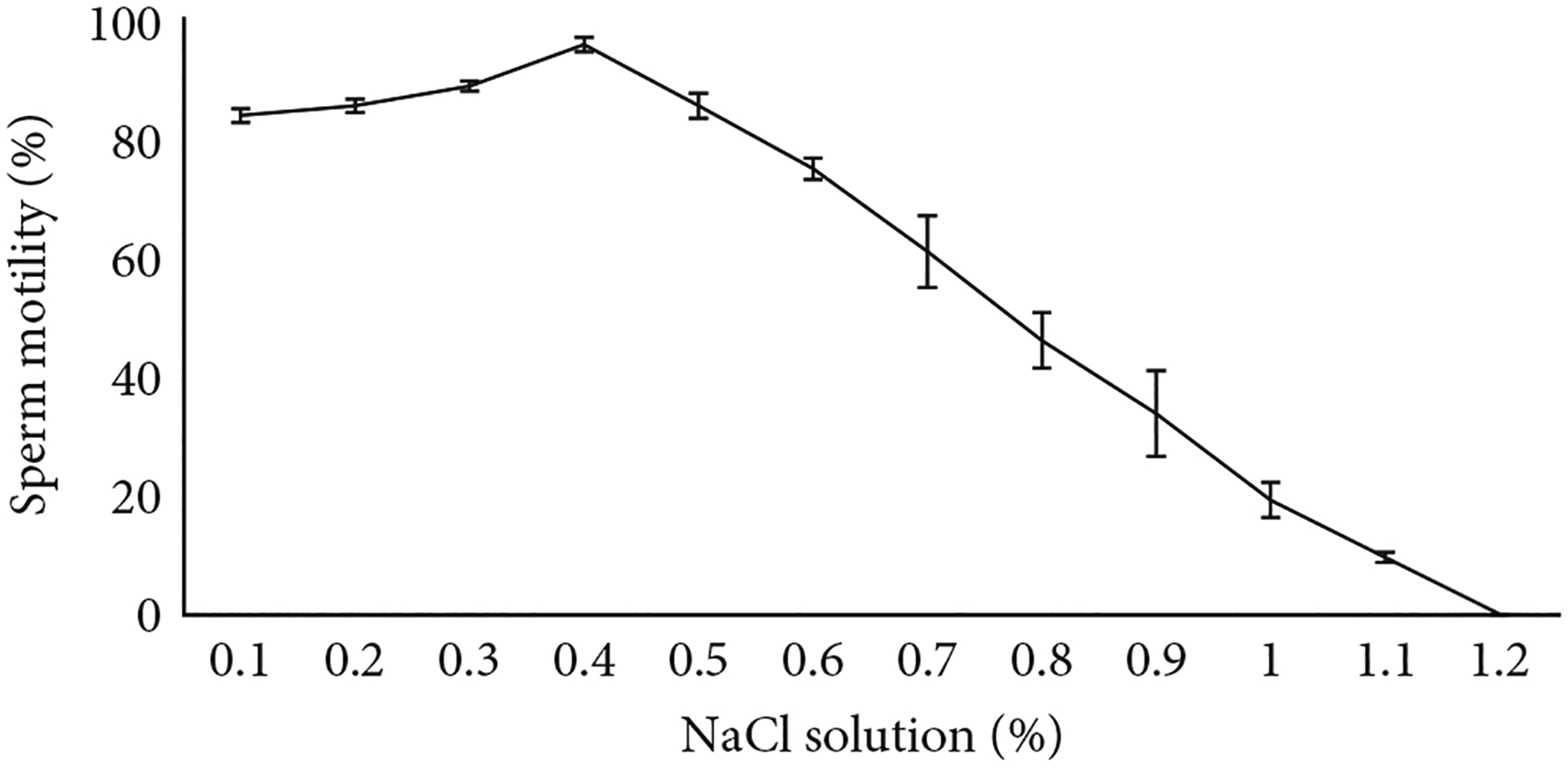
Motility of sperm of *C. idella* along an osmotic gradient of NaCl concentrations.

Activation of sperm motility decreased with increasing osmolality of NaCl solutions (Figure 1). The highest motility was observed at 128 mOsmol/kg (0.4% NaCl). After that, the motility sharply decreased with increasing osmolality and became zero at 383 mOsmol/kg (1.2% NaCl). Motility of sperm at 128 mOsmol/kg and below was stable and was considered to be complete activation. A significant difference (*p* <0:05) was observed between complete activation (128 mOsmol/kg) and inhibition (383 mOsmol/kg) of sperm.

### 3.3. Effect of Osmotic Pressure on Motility Duration.

**Figure 2:**
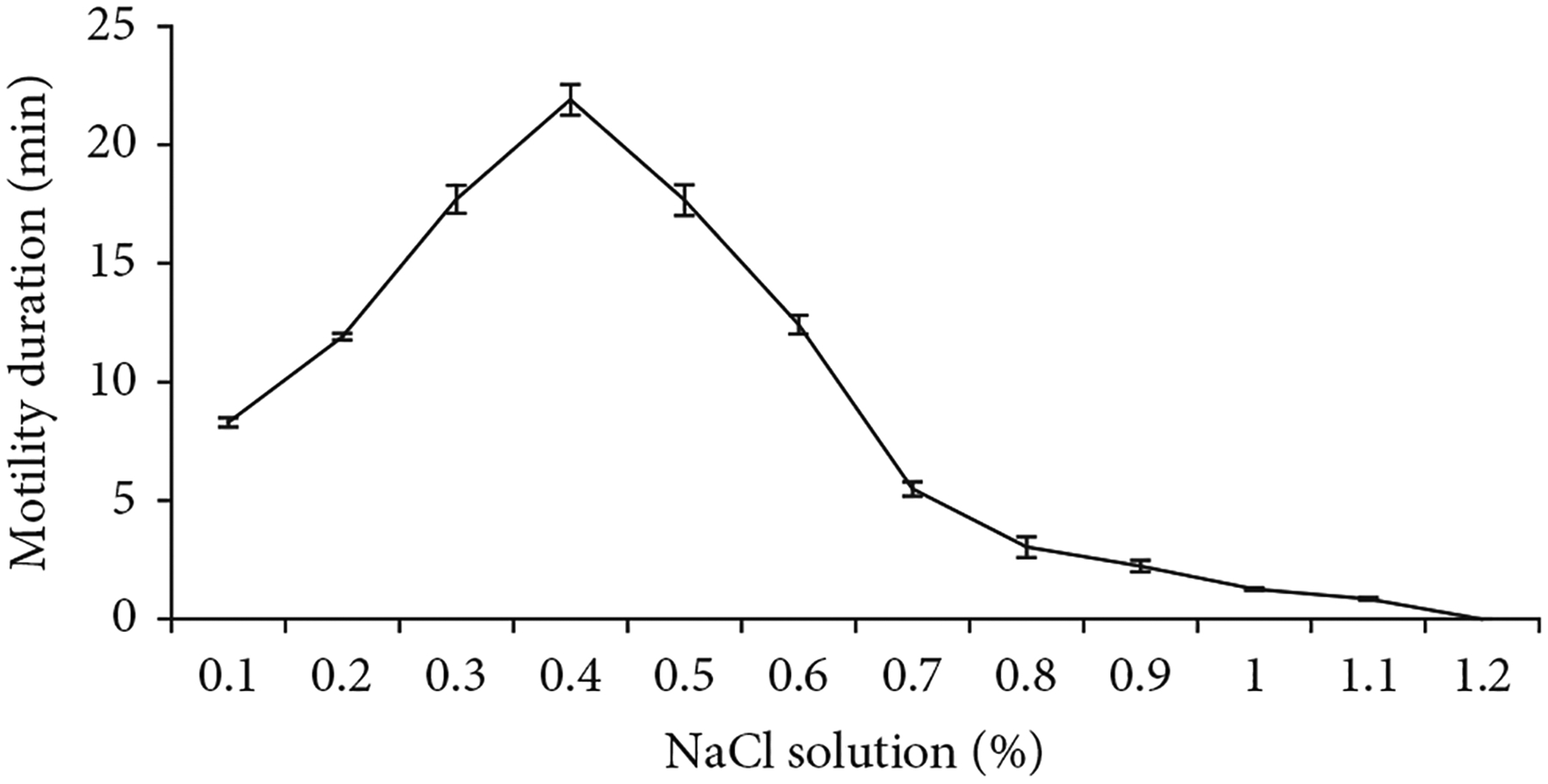
Motility duration of *C. idella* sperm at different concentrations of NaCl solution.

The motility duration of activated sperm differed at various osmolalities of NaCl solution. The initial motility duration of sperm was 8.3 ± 0.2 min at 48 mOsmol/kg (0.1% NaCl), and the highest motility duration was 21.9 ± 0.7 min at 128 mOsmol/kg (0.4% NaCl). After that, the motility duration reduced gradually with the increased osmolality of NaCl and became zero at 383 mOsmol/kg (1.2% NaCl) (Figure 2). Statistical analysis showed that the motility duration of activated sperm at 128 mOsmol/kg was significantly higher (*p* <0:05) than that of 383 mOsmol/kg.

### 3.4. Evaluation of Toxicity of Cryoprotectant to Sperm.

The motility of fresh sperm just after collection was 90% ± 1%. Sperm motility decreased with increasing cryoprotectant concentration (5%, 10%, and 15%) and incubation time (5–40 min). In Alsever’s solution, sperm incubated with 5% DMSO for 5 min showed 68% ± 2% motility, which was reduced to 55% ± 3% at 10 min and completely ceased after 35 min of incubation (Table 1). With 10% DMSO, sperm motility was as 83% ± 2% at 5 min, which decreased to 70 ± 1% at 10 min, and completely ceased at 40 min. With 15% DMSO, sperm motility was 41% ± 2% at 5 min, reduced to 32 ± 2% at 10 min, and completely ceased at 30 min. DMSO at 5%, 10%, and 15% with Alsever’s solution showed significant differences (*p* <0:05) in sperm motility through 15 min of incubation.

### 3.5. Selection of Suitable Diluent and Sperm–Diluent Ratio.

**Figure 3:**
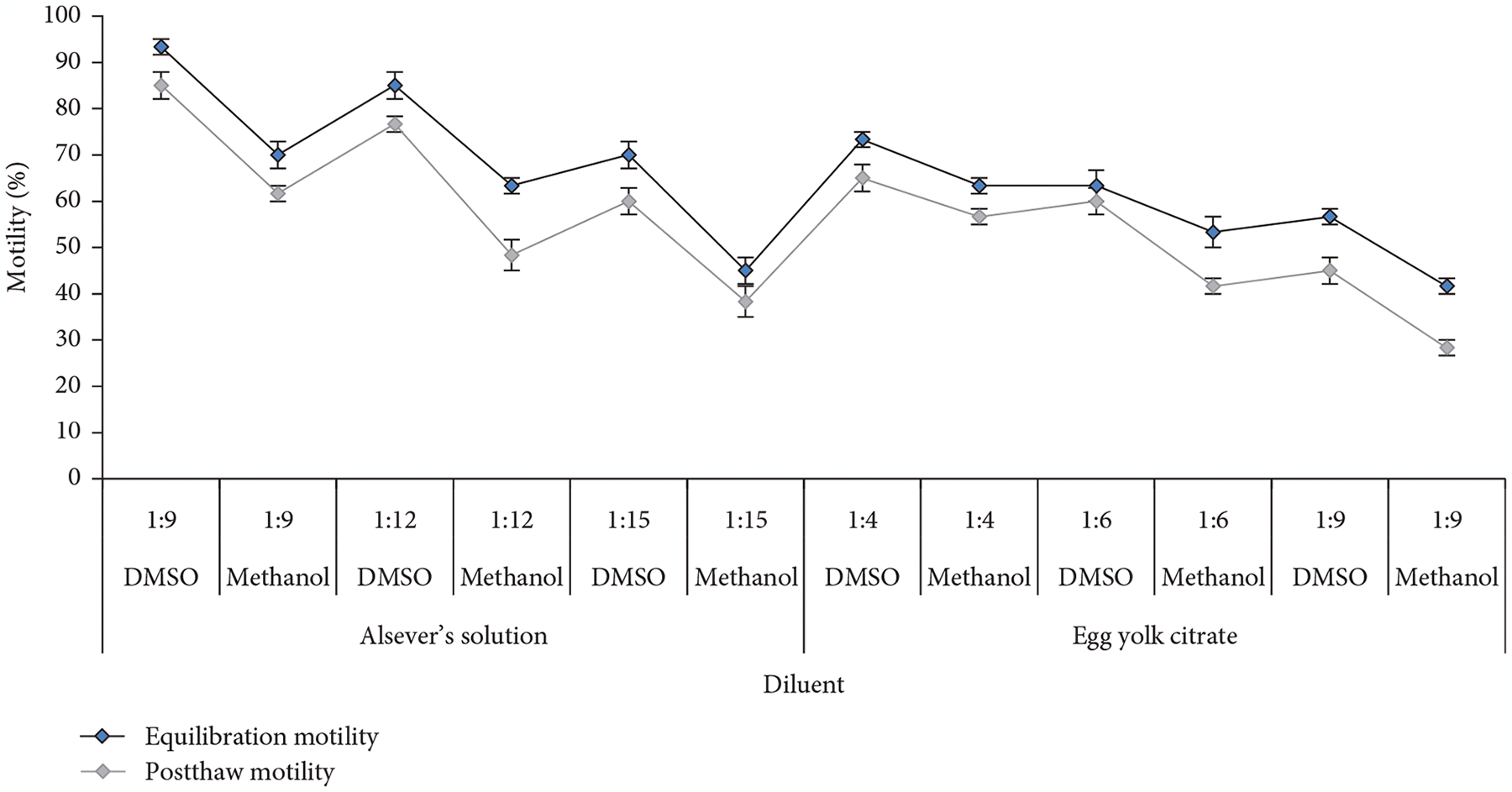
Equilibration and postthaw motility of sperm of *C. idella* in different combinations of extenders and cryoprotectants at different dilutions of sperm.

The postthaw motility was reduced from the equilibration motility in all diluents. Sperm diluted with Alsever’s solution plus DMSO at 1:9 produced the highest equilibration motility (93% ± 2%) followed by Alsever’s solution with DMSO at 1:12 (85% ± 3%). Similarly, Alsever’s solution with DMSO recorded the highest postthaw motility (85% ± 3%) at 1:9 dilution followed by (77% ± 2%) with Alsever’s solution plus DMSO at 1:12 dilution (Figure 3). In case of egg yolk citrate, DMSO at 1:4 produced the highest equilibration (73% ± 2%) and postthaw (65% ± 3%) motility compared to other combinations of extenders and cryoprotectants and dilution ratios. DMSO produced significantly (*p* <0:05) higher equilibration and postthaw motility compared to methanol with both extenders (Figure 3). Duncan’s multiple range test revealed that Alsever’s solution with DMSO would be the suitable diluent for cryopreservation of sperm of *C. idella* at a sperm diluent ratio of 1:9.

### 3.6. Determination of Suitable Equilibration Time.

**Figure 4:**
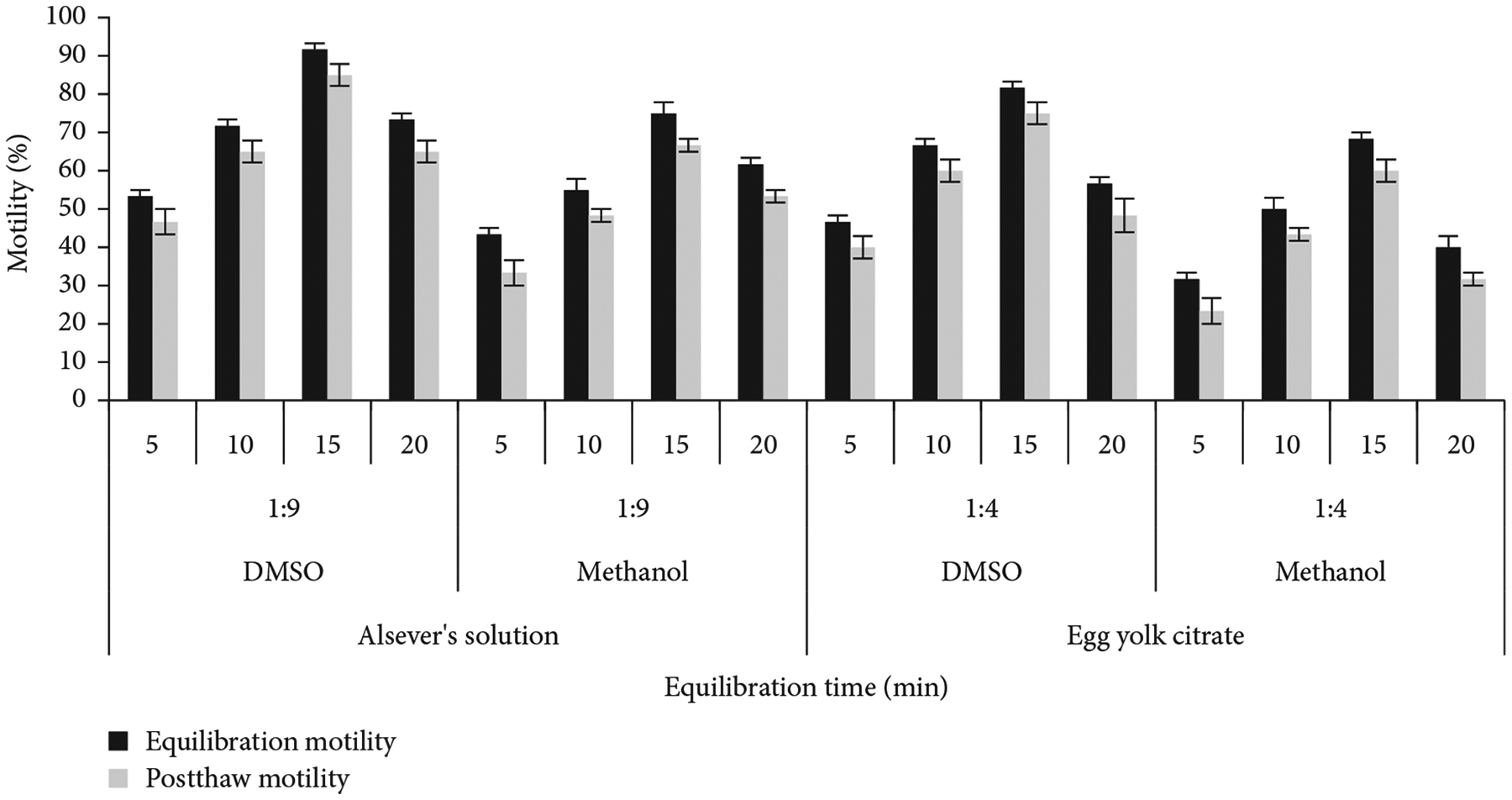
Equilibration and postthaw motility of sperm of *C. idella* equilibrated at four different periods.

Sperm were cryopreserved by maintaining four different equilibration periods (5, 10, 15, and 20 min). Sperm preserved with Alsever’s solution plus 10% DMSO recorded the highest equilibration (92% ± 2%) and postthaw (85% ± 3%) motility at a 15-min equilibration at 1:9 dilution (Figure 4). Alsever’s solution plus 10% methanol produced the highest equilibration motility (75% ± 3%) and postthaw motility (67% ± 2%) at 15-min equilibration at the same dilution.

Sperm preserved with egg yolk citrate plus 10% DMSO showed the highest equilibration (82% ± 2%) and postthaw (75% ± 3%) motility at a 15-min equilibration at 1:4 dilution. Egg yolk citrate with 10% methanol produced the highest equilibration motility (68% ± 2%) and postthaw motility (60% ± 3%) at a 15-min equilibration at the same dilution (Figure 4). An equilibration time of 15 min with Alsever’s solution and DMSO at 1:9 dilution produced significantly (*p* <0:05) the highest equilibration and postthaw motility.

### 3.7. Determination of Suitable Cooling Rate.

**Figure 5:**
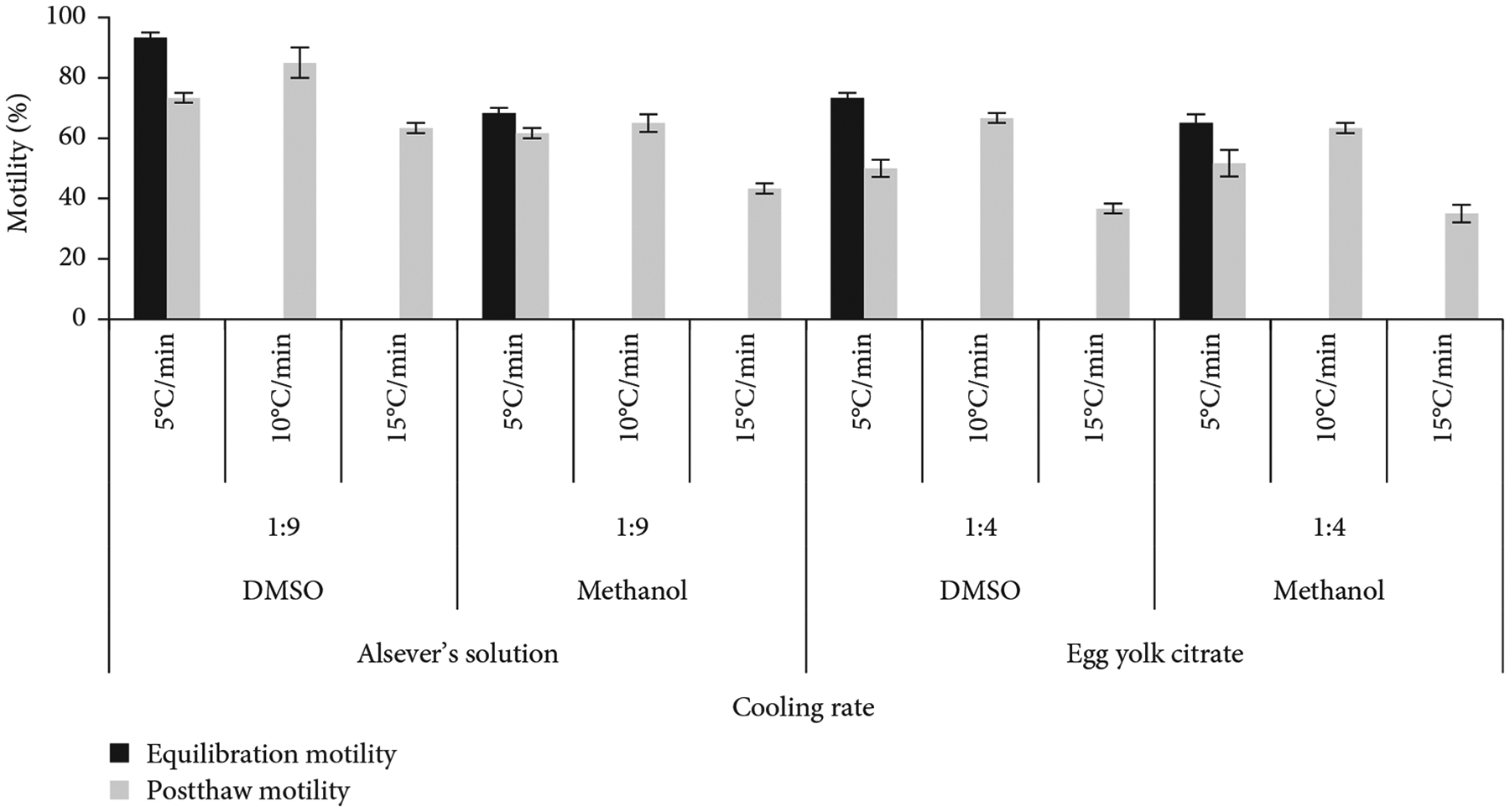
Postthaw motility of sperm of *C. idella* at different cooling rates during cryopreservation.

During cryopreservation, diluted sperm were cooled from 4 to −80°C at 5, 10, and 15°C/min. Alsever’s solution plus 10% DMSO produced the highest postthaw motility (85% ± 5%) when cooled at 10°C/min (Figure 5). Similarly, egg yolk citrate with DMSO produced the highest postthaw (67% ± 2%) motility when cooled at 10°C/min. In all the combinations of extenders and cryoprotectants, significantly (*p* <0:05) higher postthaw motility was observed at a cooling rate of 10°C/min.

### 3.8. Effects of Cryopreserved Spermatozoa on Fertilization and Hatching Rates.

**Figure 6:**
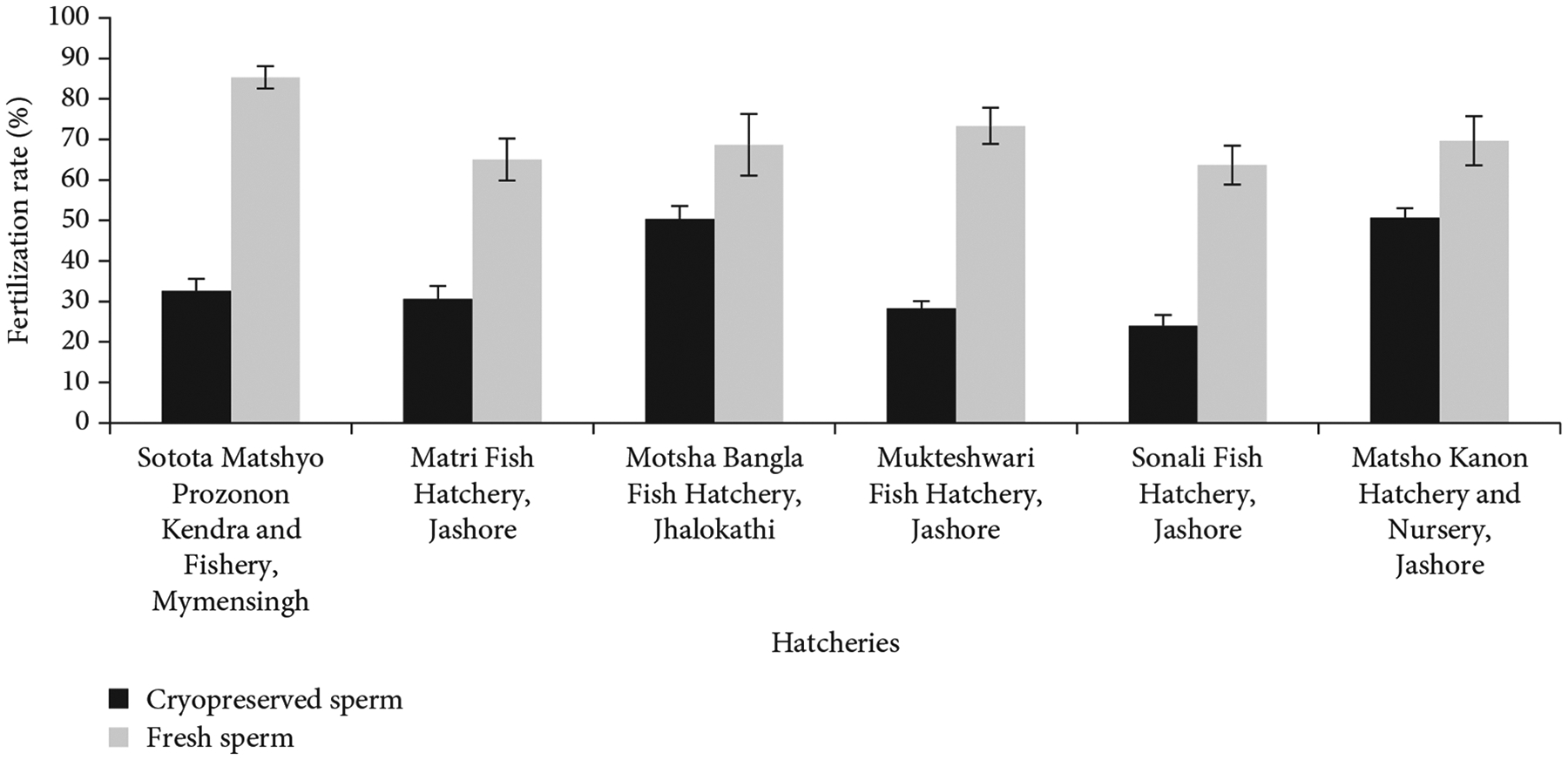
Fertilization rates of eggs of *C. idella* using cryopreserved and fresh sperm in six selected private hatcheries.

**Figure 7:**
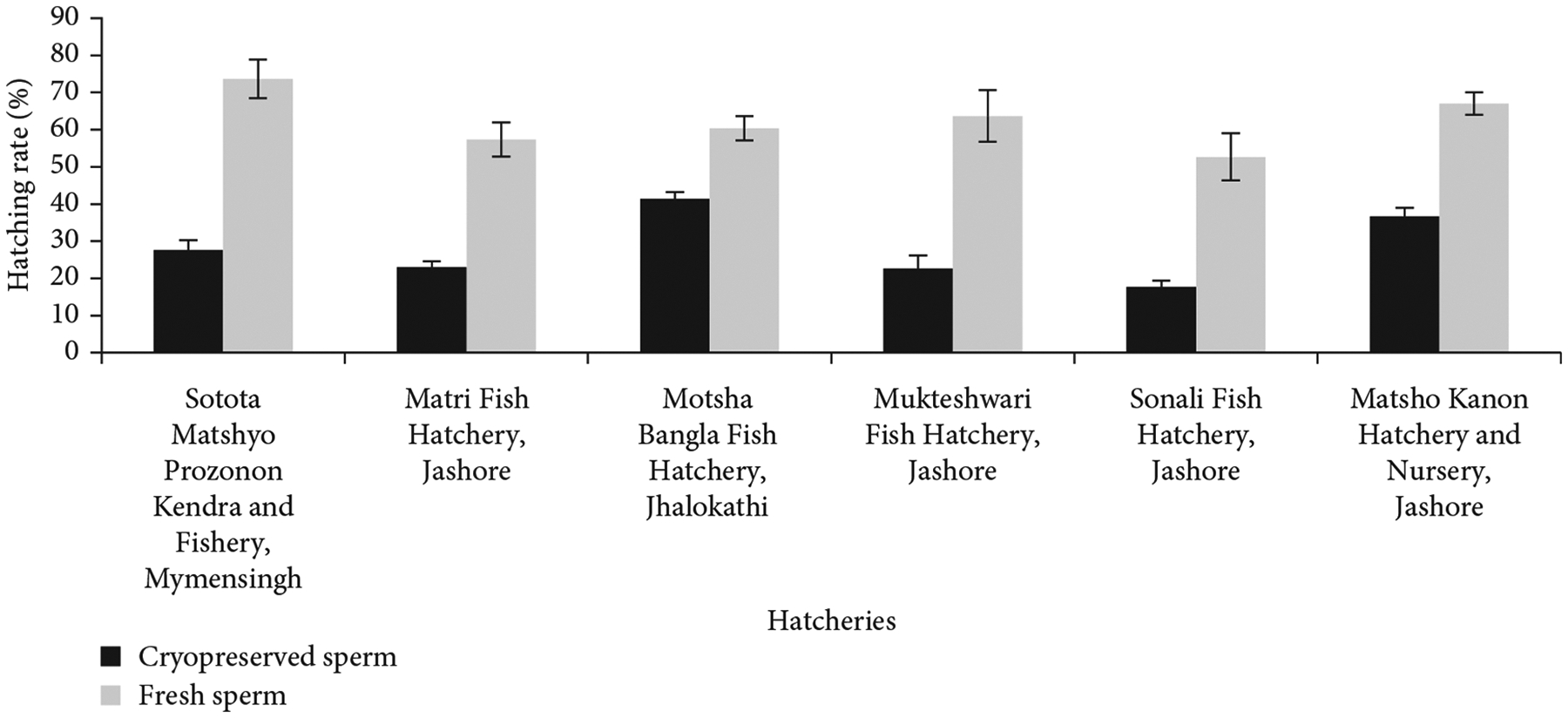
Hatching rates of eggs of *C. idella* using cryopreserved and fresh sperm in six selected private hatcheries.

Fresh and cryopreserved sperm were used to fertilize eggs. Sperm cryopreserved with Alsever’s solution plus 10% DMSO at 1:9 dilution ratio were used in hatchery-level breeding trials to evaluate fertilization and hatching. Fertilization of eggs in six hatcheries ranged from 24% ± 3% to 51% ± 2% for cryopreserved sperm and 64% ± 5% to 85% ± 3% for hatchery-origin fresh sperm (Figure 6). Hatching rates were between 18% ± 2% and 41% ± 2% for cryopreserved sperm and between 53% ± 6% and 74% ± 5% for fresh sperm in those hatcheries (Figure 7). The nominal efficiency of cryopreserved sperm and fresh sperm was noted, but since the concentration of fresh and cryopreserved sperm was not standardized, no statistical comparison was conducted between their abilities to fertilize eggs.

## 4. Discussion

During the 1960s and early 1970s, the freshwater aquaculture system of Bangladesh was completely dependent on fish seed collected from natural sources [20]. In the 1980s, artificial seed production techniques, mostly with indigenous carps, were introduced through establishment of few hatcheries in public sector. Subsequently, a large number of hatcheries have been installed throughout the country in the public and private sectors. Now hatcheries have become the main source of fish seed production (99.7%) for aquaculture [3]. However, most of the hatcheries are experiencing problems such as inbreeding, unplanned hybridization, inappropriate selection, improper broodstock management, and genetic drift. As a result, the performance of produced seeds has decreased over the years, which causes economic loss to hatchery owners, nursery operators, fry traders, and fish farmers. As such, almost 91% of fish farmers suffered from an inadequate supply of quality fish seeds during pond fish farming [21]. Assurance of good-quality seeds could resolve this problem and enhance aquaculture production. So, it is essential to take proper interventions to produce high-quality fish seeds in hatcheries to support sustainable aquaculture production of Bangladesh. Therefore, this study was aimed at using cryopreserved *C. idella* sperm to assess how the technology could be best used for seed production in selected hatcheries of four different regions of Bangladesh.

Cryopreservation of fish sperm depends on multiple factors that must be addressed to maximize their effectiveness. These include suitable combinations of extender and cryoprotectant, the dilution ratio between milt and diluent, suitable concentrations of cryoprotectant, and fertilization and hatching performance of cryopreserved sperm. The concentration of fresh sperm of *C. idella* was estimated as 2.4 ± 0.3 × 10^10^ cells/mL. This is in line with previous results of 2.1 ± 5.5 × 10^10^ cells/mL [22] and 3.0 ± 0.2 × 10^10^ cells/mL [23]. The pH of *C. idella* spermatozoa was slightly alkaline (8.3 ± 0.1) and was similar to the findings of 8.3 ± 0.2 [22] and 7.9 ± 0.1 [23].

The most typically used indicator of sperm quality is motility, which can have a high correlation with fertilization [24]. The motility of fresh sperm was found to be 90 ± 1% which was similar to 89% ± 3% [23] and 87% ± 7% [22] reported, previously. Generally, fish sperm remains immotile in the testis [25] and become motile once expelled into the aquatic environment [26–28]. Factors such as ionic composition, milt dilution ratio, medium osmolality, and temperature are thought to influence sperm motility. The activation mechanisms of sperm of various animals can differ from each other. The sperm of freshwater species are typically activated in hypotonic solutions, while in marine species, the sperm are activated in hypertonic solutions [25, 28]. Therefore, it is essential to comprehend the sperm activation process and motility before choosing an acceptable extender for the cryopreservation of sperm.

Sperm motility decreased with increasing osmolality of the activating solution (NaCl), and it was totally inhibited at 383 mOsmol/kg which agrees with findings for *Nandus nandus* [29] and *Ompok pabda* [16]. In zebrafish (*Danio rerio*), sperm motility was also found to decrease when the osmolality increased [18]. The osmolalities of the seminal plasma of most freshwater cyprinids are between 230 and 346 mOsmol/kg [30] which is closer to this species (348 mOsmol/kg). Therefore, an extender solution with an osmolality similar to seminal plasma would be appropriate for cryopreservation. Alsever’s solution, which is formulated for this study, contained osmolality of 240 mOsmol/kg, seemed to be effective for protecting sperm during storage.

Lower osmolalities of the NaCl solution allowed the activated sperm of *C. idella* to continue swimming for a longer period of time. The highest motility duration (22 ± 1 min) was found at 128 mOsmol/kg (0.4% NaCl). This may enhance the chance for interaction of sperm and eggs and consequently increase the fertilization rate. In fact, there is a conventional practice of using saline solution (0.85% NaCl solution) by most hatchery operators of Bangladesh during sperm and egg mixing assuming that it will increase fertilization rate. It is somehow relevant as we have observed that 0.4% NaCl solution yielded the highest motility and longest motility duration of sperm. Addition of 0.85% NaCl during egg–sperm mixing would have provided an osmolality closer to 128 mOsmol/kg through dilution with ovarian fluids that may facilitate higher fertilization.

A crucial factor in establishing any cryopreservation protocol is choosing the extender, the cryoprotectant, and their combination. Moreover, if there is no change in the morphological structure of the cryopreserved sperm, equilibration and postthaw motility can imply the success of cryopreservation [31]. Cryoprotectants are used to protect cells from cold shock and to reduce cell stress during cooling and freezing, but there are also different levels of toxicity to the cell. For the cryopreservation of fish sperm, there is no universal concentration and type of cryoprotectant [32]. Cryoprotectants in this study were chosen in accordance with previous studies on zebrafish (*D. rerio*) [18], rohu (*Labeo rohita*) [33], grass carp (*C. idella*) [15], and meni (*N. nandus*) [29]. In the present study, 5% and 10% DMSO with Alsever’s solution yielded higher motility during incubation times of 5 and 10 min, whereas egg yolk citrate yielded comparatively lower motility during the same incubation time with the same DMSO concentrations. DMSO at 10% concentration yielded the highest percentage of motility perhaps due to replacement of more water from the sperm cell during equilibration making less water available for intracellular ice formation. Therefore, from the suitability tests of cryoprotectant concentration, it was concluded that 10% DMSO with Alsever’s solution would be the best combination for *C. idella*.

Through this study, a number of experiments were conducted to obtain suitable conditions of basic parameters of sperm cryopreservation. However, the main goal was to establish a sperm repository of grass carp using standardized parameters. This repository would serve as a reliable source of quality sperm for seed production of the species. In Bangladesh, more than 1000 hatcheries, public and private, are being operated for producing seeds of different commercial fish species to meet the demand of farmers. Primarily, the quantity of seeds produced seems to fulfill the requirement, but the genetic quality is not adequate. Seeds produced in those hatcheries are often inferior due to genetic factors such as inbreeding, interspecific hybridization, and negative trait selection. Therefore, establishment of sperm bank or repository has become essential. Through the present study, sperm of grass carp have been cryopreserved, and a cryogenic sperm bank was established at BAU.

To determine the breeding performance of grass carp using cryopreserved sperm, a number of private hatcheries were selected in the Mymensingh, Faridpur, Jashore, and Barishal regions covering east, southwest, and central parts of Bangladesh. Breeding was conducted in seven hatcheries in three regions except Faridpur, and successful results were obtained from six hatcheries. Sperm preserved with Alsever’s solution and DMSO was successfully used in hatchery breeding trials. The fertilization and hatching rates of the eggs produced from the cryopreserved sperm were lower than those of the fresh sperm though the cryopreserved sperm indicates its potentiality in fertilizing and hatching of eggs. It is difficult to pinpoint the actual causes of low fertilization and hatching rates. However, some fundamental damage to spermatozoa during cryopreservation may happen, which limits spermatozoan function such as motility, plasma membrane integrity and functionality, ATP content, and DNA damage, ultimately causing low fertilization [34]. Similar assumptions were made by Hossain and Sarder [13] in the cryopreservation of silver carp (*Hypophthalmycthys molitrix*), [35] in Mrigal. In the current study, the highest fertilization and hatching rate with cryopreserved sperm were found 50% ± 3% and 41% ± 2%, respectively, in Motsha Bangla Fish Hatchery, Jhalokathi, whereas the lowest fertilization and hatching rate with cryopreserved sperm was found 24% ± 3% and 18% ± 2%, respectively, in Sonali Fish Hatchery, Jashore. The reasons for fluctuating fertilization and hatching rates using cryopreserved sperm between hatcheries are due to variation in quality of eggs and sperm, quality of hatchery water, breeding facilities, etc. However, the fertilization and hatching rates were nominally higher with fresh sperm in all the breeding trials as it was freshly collected and used for fertilization within a min. Sultana et al. [36] reported the best fertilization of 37.7% ± 1.76% and hatching of 28.7 ± 1.85% of common carp (*Cyprinus carpio*) using sperm processed with egg yolk citrate plus 10% DMSO compared to other diluents, which supports the results of the current investigation. The fertilization and hatching rate in the current study were also comparable to the results obtained by Muchlisin et al. [37], although they used 5% egg yolk and 5% DMSO for sperm cryopreservation in *Rasbora tawarensis*. In *Sorubim cuspicaudus*, the fertilization rate using thawed semen ranged from 42% ± 9% to 26% ± 10%, and the hatching rate varied from 23% ± 1% to 14% ± 2% when using ethylene glycol as cryoprotectant and 6% glucose and 5% skim milk powder as extender [38]. The postthaw motility of the cryopreserved sperm of *C. idella* might have an impact on fertilization and hatching as the relationship between motility and fertility is often considered to be in close proximity. The structural damage that occurred during the freezing and thawing processes was attributed to reduced fertilization and hatching rates [38]. However, in spite of having lower fertilization and hatching rate, *C. idella* fry were produced using cryopreserved sperm in the private hatcheries in Bangladesh.

Before actual breeding was done, the details of sperm cryopreservation were demonstrated to the hatchery operators. The hatchery operators also received hands-on training on seed production using cryopreserved sperm in hatcheries. They learned techniques for thawing of frozen straws, cutting of straws and mixing thawed sperm with eggs, and calculation of fertilization and hatching rates of eggs. They also engaged in rearing the produced seeds in their nursery facilities to convert them into broodstock. This would enable them to produce quality broods year after year if cryopreserved sperm were made available to them.

In Bangladesh, most of the hatchery owners or operators are not well educated, but they have willingness to receive advanced technology within a short span of time through proper training. Because the development of sperm repositories is technology dependent and requires expensive equipment and reasonable space, the government could initiate a program to establish a sperm repository in a central place as well as in carp-dominated regional areas. It would facilitate conservation of exiting germplasm and ensure a supply of required sperm to the hatcheries. However, to avoid the procurement of expensive equipment and for making cryopreservation technology more simple, open hardware technology as described by Childress, Liu, and Tiersch [39] and Tiersch et al. [40] could be applied to develop tools that are comparatively cheap and easily useable by hatchery operators.

Large-scale use of cryopreserved sperm for fish seed production in public and private hatcheries scattered over Bangladesh will necessitate the establishment of cryogenic sperm repositories. This will ensure production of quality seeds and development of quality broodstocks. Most of the hatcheries have the basic facilities required for adopting the technology, and hatchery owners are eager to accept new innovations. This positive attitude of the hatchery owners of Bangladesh demands appreciation.

## 5. Conclusion

Cryopreservation of *C. idella* sperm was demonstrated to provide satisfactory results in farmers’ installations. Although *C. idella* seeds were produced using cryopreserved sperm in hatcheries, there is room for improvement of fertilization and hatching rates.

## Figures and Tables

**Table 1:** Motility of C. idella sperm at different concentrations of cryoprotectants and incubation times.

Extender	Cryoprotectant	Sperm motility (%) at different incubation times (min)									
		Cryoprotectant concentration (%)	Initial (0)	5	10	15	20	25	30	35	40
Alsever’s solution	DMSO	5	83 ± 2	68 ± 2	55 ± 3	44 ± 2	26 ± 2	12 ± 2	2 ± 2	0 ± 0	—
		10	93 ± 2	83 ± 2	70 ± 1	48 ± 1	29 ± 2	20 ± 3	10 ± 2	4 ± 4	0 ± 0
		15	62 ± 2	41 ± 2	32 ± 2	15 ± 3	7 ± 2	2 ± 2	0 ± 0	—	—
	Methanol	5	68 ± 2	55 ± 3	40 ± 3	20 ± 3	7 ± 3	0 ± 0	0 ± 0	—	—
		10	60 ± 3	50 ± 3	40 ± 3	15 ± 3	5 ± 3	2 ± 2	0 ± 0	—	—
		15	40 ± 3	32 ± 4	20 ± 3	10 ± 3	2 ± 2	0 ± 0	0 ± 0	—	—
Egg yolk citrate	DMSO	5	69 ± 2	58 ± 2	44 ± 2	30 ± 2	15 ± 2	8 ± 2	1 ± 1	—	—
		10	59 ± 2	54 ± 2	43 ± 2	40 ± 3	18 ± 1	7 ± 2	4 ± 2	—	—
		15	49 ± 3	36 ± 1	26 ± 1	18 ± 3	8 ± 1	3 ± 2	0 ± 0	—	—
	Methanol	5	58 ± 1	49 ± 2	38 ± 1	28 ± 2	15 ± 0	8 ± 1	2 ± 2	—	—
		10	45 ± 1	39 ± 2	28 ± 2	23 ± 1	13 ± 2	3 ± 2	0 ± 0	—	—
		15	37 ± 2	24 ± 2	17 ± 2	7 ± 2	0 ± 0	—	—	—	—

*Note:* Data are presented as mean ± SE.

Abbreviation: DMSO, dimethyl sulfoxide.

## Data Availability

Data will be made available on request.

## References

[1] Yang G, Zhao W, Qin C, , “Molecular Identification of Grass Carp igfbp2 and the Effect of Glucose, Insulin, and Glucagon on igfbp2 mRNA Expression,” Fish Physiology and Biochemistry 46, no. 4 (2020): 1469–1482. 32323051 10.1007/s10695-020-00804-w

[2] Rahman AKA, “Freshwater Fishes of Bangladesh,” (Dhaka, Bangladesh: Zoological Society of Bangladesh: 263, 2nd edition, 2005).

[3] DoF, “Yearbook of Fisheries Statistics of Bangladesh, 2021–22, Fisheries Resources Survey System (FRSS), Department of Fisheries,” Ministry of Fisheries and Livestock 39 (2022), 139.

[4] Bodenstein S, Tiersch TR, Hossain MAR, , “A Cryopreserved Sperm Repository Strategy for WorldFish Genetically Improved Carp,” 2023, https://hdl.handle.net/20.500.12348/5501.

[5] Tiersch TR, “Process Pathways for Sperm Cryopreservation Research, Application, and Commercialization,” in Cryopreservation in Aquatic Species, eds. Tiersch TR and Green CC, (World Aquaculture Society, Baton Rouge, Louisiana, USA, 2nd edition, 2011): 646–671.

[6] Torres L and Tiersch TR, “Addressing Reproducibility in Cryopreservation, and Considerations Necessary for Commercialization and Community Development in Support of Genetic Resources of Aquatic Species,” Journal of the World Aquaculture Society 49, no. 4 (2018): 644–663. 30467453 10.1111/jwas.12541 PMC6241537

[7] Khoshoo TN, “Conservation of India’s Endangered Mega Animals: Tiger and Lion,” Current Science 73 (1997): 830–842.

[8] Tiersch TR, Huiping Y, Jenkins JA, and Dong Q, “Sperm Cryopreservation in Fish and Shellfish,” Society of Reproduction and Fertility Supplement 65 (2007): 493–508. 17644987

[9] Tiersch TR, Figiel CR, Wayman WR, Williamson JH, Carmichael GJ, and Gorman TO, “Cryopreservation of Sperm of the Endangered Razorback Sucker,” Transactions of the American Fisheries Society 127, no. 1 (1998): 95–104.

[10] Lubzens E, Daube N, Pekarsky I, , “Carp (*Cyprinus carpio* L.) Spermatozoa Cryobanks-Strategies in Research and Application,” Aquaculture 155, no. 1–4 (1997): 13–30.

[11] Suquet M, Dreanno C, Fauvel C, Cosson I, and Billard R, “Cryopreservation of Sperm in Marine Fish,” Aquaculture Research 31, no. 3 (2000): 231–243.

[12] Khan NS, Sarder MRI, Faroque MAA, and Mollah MFA, “Standardization of Sperm Cryopreservation Techniques of Indian Major Carp Rohu (*Labeo rohita*, Hamilton 1822),” International Journal of Fisheries and Aquatic Studies 2 (2015): 175–181.

[13] Hossain MS and Sarder MRI, “Cryogenic Freezing of Silver Carp Spermatozoa for Conservation of Gene Pool,” Progressive Agriculture 20, no. 1–2 (2013): 99–106.

[14] Rahman MM, Ali MR, Sarder MRI, Mollah MFA, and Khan NS, “Development of Sperm Cryopreservation Protocol of Endangered Spiny Eel, *Mastacembelus armatus* (Lacepede 1800) for Ex-Situ Conservation,” Cryobiology 73, no. 3 (2016): 316–323. 27746166 10.1016/j.cryobiol.2016.10.004

[15] Sarder MRI, Hossain MA, and Hossain MS, “Cryofreezing of Grass Carp (*Ctenopharyngodon idella*) Spermatozoa for Ex-Situ Conservation,” Progressive Agriculture 21, no. 1–2 (2010): 141–150.

[16] Sarder MRI, Saha SK, and Sarker MFM, “Cryopreservation of Sperm of an Indigenous Endangered Fish, Pabda (*Ompok pabda*, Hamilton–Buchanan, 1822),” North American Journal of Aquaculture 75, no. 1 (2013): 114–123.

[17] Nahiduzzaman M, Hassan MM, Khanam UH, Mamun SNA, Hossain MAR, and Tiersch TR, “Sperm Cryopreservation of the Critically Endangered Olive Barb (Sarpunti, *Puntius sarana*) (Hamilton, 1822),” Cryobiology 62 (2011): 62–67. 21168401 10.1016/j.cryobiol.2010.12.004

[18] Yang H, Carmichael C, Varga ZM, and Tiersch TR, “Development of a Simplified and Standardized Protocol with Potential for High-Throughput for Sperm Cryopreservation in Zebrafish *Danio rerio*,” Theriogenology 68, no. 2 (2007): 128–136. 17544099 10.1016/j.theriogenology.2007.02.015 PMC2676789

[19] Kainin S, Ponchunchoovong S, Imsilp U, and Singsee S, “Cryopreservation of Mekong Catfish, *Pangasius bocourti* Sauvage, 1880 Spermatozoa,” Aquaculture Research 45, no. 5 (2014): 859–867.

[20] Parvez MS, Rahman MA, Hasan MJ, , “Role of Hatchery on Fish Seed Production in Patuakhali District of Bangladesh: An Overview,” International Journal of Chemical, Environmental & Biological Sciences 6 (2018): 1–7.

[21] Das M, Islam MR, Akter T, Kawser AQMR, and Mondal MN, “Present Status, Problems and Prospect of Fish Farming at Gazipur Sadar Upazila in Bangladesh,” Progressive Agriculture 29, no. 1 (2018): 53–63.

[22] Dang TM, Pham MA, Pham AT, and Lee KJ, “Effects of Dimethyl-Sulfocide on Sperm Cryopreservation of Grass Carp (*Ctenopharyngodon idella*),” Journal of Aquaculture 19 (2006): 52–56.

[23] Verma DK, Routray P, Dash C, Dasgupta S, and Jena JK, “Physical and Biochemical Characteristics of Semen and Ultrastructure of Spermatozoa in Six Carp Species,” Turkish Journal of Fisheries and Aquatic Sciences 9 (2009): 67–76.

[24] Rurangwa E, Kime DE, Ollevier F, and Nash JP, “The Measurement of Sperm Motility and Factors Affecting Sperm Quality in Cultured Fish,” Aquaculture 234, no. 1–4 (2004): 1–28.

[25] Morisawa M and Suzuki K, “Osmolality and Potassium Ion: Their Roles in Initiation of Sperm Motility in Teleosts,” Science 210, no. 4474 (1980): 1145–1147. 7444445 10.1126/science.7444445

[26] Billard R and Cosson MP, “Some Problems Related to the Assessment of Sperm Motility in Freshwater Fish,” Journal of Experimental Zoology 261, no. 2 (1992): 122–131.

[27] Alavi SMH and Cosson J, “Sperm Motility in Fishes. II. Effects of Ions and Osmolality: A Review,” Cell Biology International 30, no. 1 (2006): 1–14. 16278089 10.1016/j.cellbi.2005.06.004

[28] Yang H, Hazlewood L, Walter RB, and Tiersch TR, “Effect of Osmotic Immobilization on Refrigerated Storage and Cryopreservation of Sperm from a Viviparous Fish, the Green Swordtail *Xiphophorus helleri*,” Cryobiology 52, no. 2 (2006): 209–218. 16375884 10.1016/j.cryobiol.2005.11.002 PMC5593140

[29] Sarder MRI, Sarker MFM, and Saha SK, “Cryopreservation of Sperm of an Indigenous Endangered Fish Species *Nandus nandus* (Hamilton, 1822) for Ex-Situ Conservation,” Cryobiology 65, no. 3 (2012): 202–209. 22750204 10.1016/j.cryobiol.2012.06.004

[30] Alavi SMH and Cosson J, “Sperm Motility in Fishes. I. Effects of Temperature and pH: A Review,” Cell Biology International 29, no. 2 (2005): 101–110. 15774306 10.1016/j.cellbi.2004.11.021

[31] Akcay E, Bozkurt Y, Secer S, and Tekin N, “Cryopreservation of Mirror Carp Semen,” Turkish Journal of Veterinary and Animal Sciences 28 (2004): 837–843.

[32] Gwo JC, “Cryopreservation of Sperm of Some Marine Fishes,” in Cryopreservation in Aquatic Species, eds. Tiersch TR and Mazik PM, (World Aquaculture Society, Baton Rouge, LA, 2000): 138–160.

[33] Sarder MRI, Rahman AKMA, Samad MS, Nazrul KMS, and Bhuiyan MKJ, “Cryopreservation of Sperm of *Labeo rohita* (Hamilton, 1822) and Its Use in Fertilization and Hatching of Eggs,” Progressive Agriculture 22, no. 1–2 (2011): 123–137.

[34] Cabrita E, Sarasquete S, Martinez-Paramo S, , “Cryopreservation of Fish Sperm: Applications and Prospective,” Journal of Applied Ichthyology 26, no. 5 (2010): 623–635.

[35] Sarder MRI, Rafiquzzaman SM, and Islam MF, “Cryopreservation of Spermatozoa of Mrigal, *Cirrhinus cirrhosus* With a View to Minimize Inbreeding and Hybridization,” Journal of the Bangladesh Agricultural University 7, no. 1 (2009): 211–218.

[36] Sultana M, Nahiduzzaman M, Hassan MM, Khanam MUH, and Hossain MAR, “Fertility of Cryopreserved Common Carp (*Cyprinus carpio*) Spermatozoa,” University Journal of Zoology, Rajshahi University 28 (2010): 51–55.

[37] Muchlisin ZA, Sarah PI, Aldila DF, , “Effect of Dimethyl Sulfoxide (DMSO) and Egg Yolk on Sperm Motility, Fertility and Hatching Rates of Depik *Rasbora tawarensis* (Pisces: Cyprinidae) Eggs After Short-Term Cryopreservation,” Aquaculture Research 51, no. 4 (2020): 1700–1705.

[38] Atencio-García V, Padilla-Izquierdo D, Robles-González J, Prieto-Guevara M, Pardo-Carrasco S, and Espinosa-Araujo J, “Damage to *Sorubim cuspicaudus* Sperm Cryopreserved With Ethylene Glycol,” Animals 13, no. 2 (2023): 235. 36670775 10.3390/ani13020235 PMC9854978

[39] Childress WM, Liu Y, and Tiersch TR, “Design, Alpha Testing, and Beta Testing of a 3-D Printed Open-Hardware Portable Cryopreservation Device for Aquatic Species,” Journal of Applied Aquaculture 35, no. 1 (2023): 213–236. 36777239 10.1080/10454438.2021.1955805 PMC9909779

[40] Tiersch CJ, Liu Y, Tiersch TR, and Monroe WT, “3-D Printed Customizable Vitrification Devices for Preservation of Genetic Resources of Aquatic Species,” Aquacultural Engineering 90 (2020): 102097. 32831431 10.1016/j.aquaeng.2020.102097 PMC7434064

